# *Brassica oleracea* L. var. *italica* Aquaporin Reconstituted Proteoliposomes as Nanosystems for Resveratrol Encapsulation

**DOI:** 10.3390/ijms25041987

**Published:** 2024-02-06

**Authors:** Lucia Yepes-Molina, José A. Teruel, Urban Johanson, Micaela Carvajal

**Affiliations:** 1 Aquaporins Group, Centro de Edafologia y Biologia Aplicada del Segura (CEBAS), CSIC, Campus de Espinardo, E-30100 Murcia, Spain; lyepes@cebas.csic.es; 2 Department of Biochemistry and Molecular Biology, Facultad de Veterinaria, Universidad de Murcia, E-30100 Murcia, Spain; teruel@um.es; 3 Division of Biochemistry and Structural Biology, Center for Molecular Protein Science, Department of Chemistry, Lund University, P.O. Box 124, SE-221 00 Lund, Sweden

**Keywords:** aquaporin, broccoli, membrane transporter reconstitution, proteoliposomes, nanocarrier

## Abstract

Aquaporins (AQPs), membrane proteins responsible for facilitating water transport, found in plant membrane vesicles (MV), have been related to the functionality and stability of MV. We focused on AQPs obtained from broccoli, as they show potential for biotechnological applications. To gain further insight into the role of AQPs in MV, we describe the heterologous overexpression of two broccoli AQPs (*BoPIP1;2* and *BoPIP2;2*) in *Pichia pastoris*, resulting in their purification with high yield (0.14 and 0.99 mg per gram cells for BoPIP1;2 and BoPIP2;2). We reconstituted AQPs in liposomes to study their functionality, and the size of proteoliposomes did not change concerning liposomes. BoPIP2;2 facilitated water transport, which was preserved for seven days at 4 °C and at room temperature but not at 37 °C. BoPIP2;2 was incorporated into liposomes to encapsulate a resveratrol extract, resulting in increased entrapment efficiency (EE) compared to conventional liposomes. Molecular docking was utilized to identify binding sites in PIP2s for resveratrol, highlighting the role of aquaporins in the improved EE. Moreover, interactions between plant AQP and human integrin were shown, which may increase internalization by the human target cells. Our results suggest AQP-based alternative encapsulation systems can be used in specifically targeted biotechnological applications.

## 1. Introduction

Aquaporins (AQPs), transmembrane proteins with an essential role in biological functions, primarily regulate water transport and maintain homeostasis through membrane water permeability adjustment [1]. These proteins are found in membrane vesicles (MV) isolated from natural sources, including broccoli, studied by our group [2,3]. These MV have potential applications in cosmetics and pharmacology, as they interact with human cell membranes and enhance bioactive compound uptake [4]. AQPs contribute to MV stability and interact with bioactive compounds, improving encapsulation [2,4]. Despite promising applications, aspects like AQPs’ role in vesicle stability require further exploration.

Initially used as in vitro membrane models, liposomes have gained traction due to their biocompatibility, biodegradability, and ability to encapsulate hydrophilic and lipophilic compounds [5,6]. This versatility has extended their use to carrying unstable compounds like natural extracts. Whereas liposomes as nanocarriers are well-studied, proteoliposomes (liposomes with proteins) remain relatively unexplored, presenting a wide-open field for research. Proteins could give more stability to the nanosystem and, specifically, AQPs, could improve the encapsulation because of their interaction with bioactives [3]. Hence, AQPs-containing proteoliposomes stand as a promising avenue to delve into MV stability and offer a viable nanocarrier solution.

The most efficient method to obtain pure membrane proteins is heterologous expression in the methylotrophic yeast *Pichia pastoris (renamed Komagataella phaffii)* [7,8]. Although this system provides high yields, different factors may influence recombinant expression levels and subsequent protein purification; therefore, it is necessary to develop a custom process for each protein of interest. Factors conditioning the level of gene expression are the properties of the nucleotide sequence, the mode of insertion of the sequence into the genome, or the culture conditions. To obtain the highest protein yields, the insertion of multiple copies of recombinant genes must be achieved [9,10]. The strategy is to screen for different levels of antibiotic resistance, as this will be correlated with the number of plasmids inserted. Regarding the protein purification, it is necessary to keep the protein in solution. For this, detergents are mandatory, and the selection of detergent is a critical step since the detergent properties will affect, on the one hand, the detergent removal efficiency and, on the other hand, the stability of proteins. Purified AQPs reconstituted into liposomes is one of the most used strategies to study different functionalities [11,12], but these studies could also bring different biotechnological results, such as AQPs reconstituted in liposomes as water purification filters [13].

In the fields of cosmetics and pharmaceuticals, using natural sources to obtain bioactive compounds has gained significant interest. Phenolic extracts like resveratrol-enriched extract are notable for their antioxidant and anti-inflammatory properties. However, their limited water solubility and bioavailability can hinder their effectiveness [14]. Encapsulating these extracts in liposomes provides a solution to overcome these challenges [15]. Efficient release of these encapsulated contents into target cells is crucial, highlighting the role of liposome-cell interaction. Membrane proteins, like integrins, are key for internalization [16], are responsible for internalization of exovesicles, and there is evidence suggesting that human AQP2 is involved in cell-cell adhesion through its interactions with integrins [17]. Thus, exploring the interaction between AQPs and integrins is an intriguing research direction, as incorporating AQPs into liposomes may facilitate the binding of proteoliposomes to cells.

Considering this background, the primary objective of this study is to investigate the functionality and properties of two AQPs from broccoli (BoPIP1;2 and BoPIP2;2). Firstly, we describe the successful overexpression of these proteins in *P. pastoris* and their purification. Subsequently, we evaluate the functionality of the reconstituted AQPs in liposomes and conduct a size stability assay. In addition, we explore the potential application of BoPIP2;2 proteoliposomes as carriers for a resveratrol extract. To determine the role of AQPs in the encapsulation capacity of proteoliposomes and their interaction with target human cells, we perform molecular docking assays.

## 2. Results

### 2.1. BoPIP1;2 and BoPIP2;2 Production in P. pastoris: Cell Yield and Membrane Recovery

**Figure 1:**
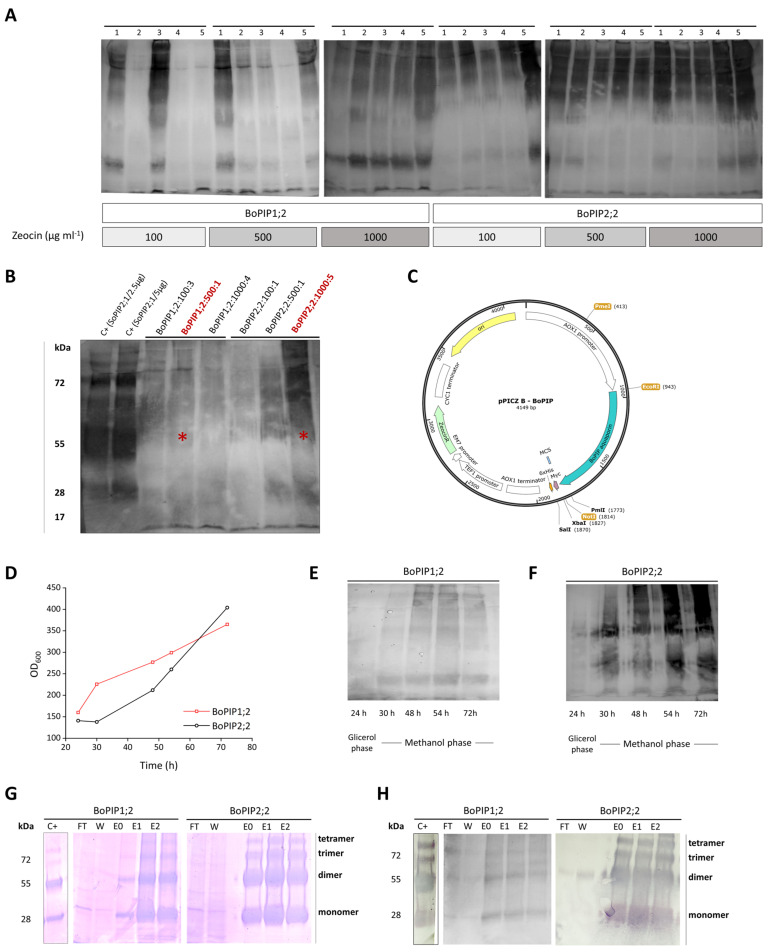
Optimization of BoPIP1;2 and BoPIP2;2 purification from *Pichia pastoris*. (**A**) Western-blot with crude cell extract of five clones from each zeocin level (100, 500, and 1000 µg zeocin mL^−1^) for *BoPIP1;2* and *BoPIP2;2*. (**B**) Western-blot with the three clones exhibiting the highest expression. Asterisks indicate the selected clones for further trials. (**C**) pPICZB vector scheme with a BoPIP encoding insert. (**D**) OD600 of samples from the fermenter at different time points. (**E**,**F**) Western-blot for BoPIP1;2 and BoPIP2;2 of crude cell lysates at different time points. (**G**) Coomassie-stained SDS-PAGE gel and (**H**) western-blots showing the positive control (C+), flow-through (FT), wash fractions (W), and elution fractions (E0, E1, and E2) obtained from the Ni-NTA His trap column during the protein purification process.

To obtain purified BoPIP1;2 and BoPIP2;2, the proteins were transformed into the yeast *P. pastoris* using the construction outlined in Figure 1C. To optimize the production of purified proteins at a small scale before proceeding to large-scale production, various parameters were examined. To screen for high-yielding clones, five clones for each construct and each zeocin concentration were analyzed by immunoblotting (Figure 1A). The best clones were selected and compared for expression levels in the same western-blot (Figure 1B). Based on the expression levels of each isoform, the clone with the highest expression was chosen for further experiments. In the case of *BoPIP1;2*, the best results were obtained with a clone selected at 500 µg zeocin mL^−1^. For *BoPIP2;2*, the expression level showed a positive correlation with the zeocin concentration, and the highest expression was achieved with 1000 µg mL^−1^ zeocin. The selected clones were produced on a large scale. The cell biomass was monitored at different time points, and after 72 h, a similar amount of biomass was reached for both isoforms (Figure 1D–F). At the end of fermentation, cell and protein yields were calculated for each AQP isoform overexpressed in *P. pastoris*. 1.5 L of culture gave 590 and 655 g of cells harvested 72 h after induction for *BoPIP1;2* and *BoPIP2;2*, respectively. Regarding protein yield, from 1.5 L of culture, 4100 and 6300 mg of total membrane proteins were obtained, corresponding to 7 and 10 mg per gram of cells for BoPIP1;2 and BoPIP2;2, respectively.

### 2.2. Membrane Protein Solubilization and AQP Purification

A solubilization screen was conducted to determine the most effective detergent for large-scale solubilization. Among the tested detergents, n-Octyl-β-D-glucoside (OG) demonstrated the best solubilization efficiency for both proteins (Figure S1). The solubilized proteins were then purified using affinity chromatography through the added His-tag at the C-terminus of the recombinant BoPIP1;2 and BoPIP2;2. The purification process was checked by Coomassie-stained and Western-blot (Figure 1G,H), which demonstrated the enrichment of BoPIP1;2 and BoPIP2;2 in the elution fractions. Approximately 0.14 mg and 0.99 mg of pure proteins per gram of cells were obtained for BoPIP1;2 and BoPIP2;2, respectively. Both purified AQPs exhibited a similar pattern: monomers, dimers, trimers, and tetramers.

### 2.3. Reconstitution of BoPIP1;2 and BoPIP2;2 in Liposomes

BoPIP1;2 and BoPIP2;2 were reconstituted in liposomes, and the resulting proteoliposomes and empty liposomes were characterized (Table 1). Sizes between 255 and 296 nm and polydispersity index (PDI) of 0.32–0.34 were obtained without significant differences between samples. To assess the functionality of the purified proteins, water channel activity was determined using stopped-flow spectrophotometry. BoPIP2;2 proteoliposomes showed an increase in both rate constants and Pf compared to empty liposomes, indicating that BoPIP2;2 is functional and capable of channeling water. No significant differences were found between BoPIP1;2 proteoliposomes and empty liposomes.

**Figure 2:**
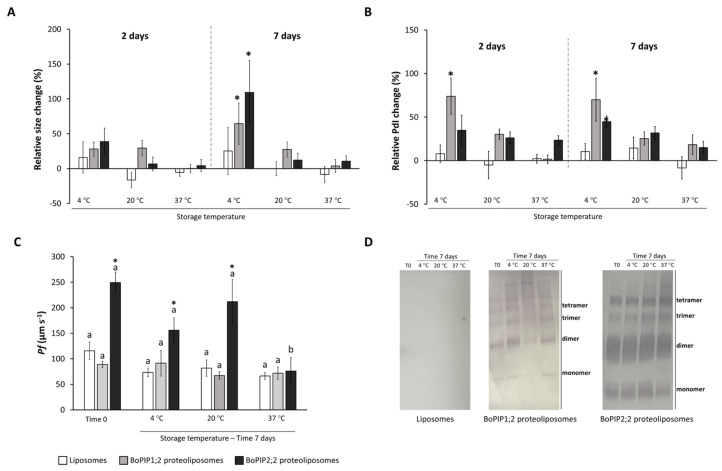
Stability and functionality of liposomes and proteoliposomes over time. (**A**) Relative change in size and (**B**) polydispersity index (PDI) of empty liposomes, BoPIP1;2 proteoliposomes, and BoPIP2;2 proteoliposomes compare to time 0 during storage for two and seven days at 4 °C, 20 °C, and 37 °C. Asterisks indicate significant differences in each sample at each time and temperature compared to the initial time. (**C**) Osmotic water permeability (Pf) and (**D**) western-blots of liposomes, BoPIP1;2 proteoliposomes, and BoPIP2;2 proteoliposomes analyzed after storage for seven days at different temperatures. Different letters indicate significant differences among conditions for each sample according to one-way ANOVA followed by Tukey-HDS test (*p* < 0.05). Asterisks (*) indicate significant differences between both BoPIP1;2 and BoPIP2;2 proteoliposomes and empty liposomes for each condition according to Student *t*-test (*p* < 0.05). Data are mean ± SE (n = 3).

Furthermore, a stability assay was conducted to assess the behavior of liposomes and proteoliposomes over time at different temperatures. Liposomes and proteoliposomes did not change their size after two days of storage. However, significant size changes were observed in both types of proteoliposomes after seven days of storage at 4 °C. In contrast, both liposomes and proteoliposomes maintained their size when stored at higher temperatures (Figure 2A). An increase in PDI was observed after seven days of storage at 4 °C, specifically for proteoliposomes but not for liposomes. Besides, this increase in PDI was also observed in BoPIP1;2 proteoliposomes after two days at 4 °C (Figure 2B).

The functionality of AQPs was also assessed after seven days of storage (Figure 2C). Initially, both liposomes and BoPIP1;2 proteoliposomes had the same Pf, around 100 µm s^−1^, and both samples maintained these values of Pf in all tested conditions after seven days. BoPIP2;2 proteoliposomes had a higher Pf (250 µm s^−1^), which remained unchanged after seven days at 4 °C and 20 °C, but a significant decrease was observed at 37 °C. Furthermore, the protein levels and the arrangement pattern of AQPs (monomers, dimers, trimers, and tetramers) were analyzed (Figure 2D). No significant changes in protein abundance of BoPIP1;2 were observed at 4 °C and 37 °C and at any condition in the case of BoPIP2;2. Regarding the AQP arrangement, no significant differences were observed.

### 2.4. Encapsulation of Resveratrol Extract in BoPIP2;2 Proteoliposomes

**Figure 3:**
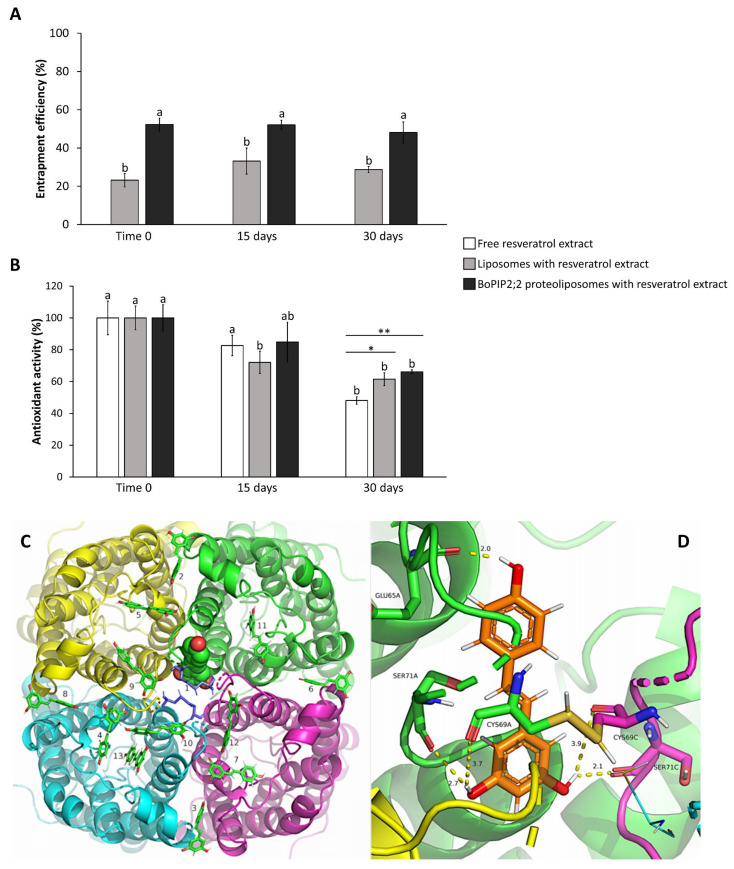
Resveratrol encapsulation in liposomes and proteoliposomes and resveratrol-aquaporin docking. (**A**) Entrapment efficiency (%) of resveratrol extract in liposomes and BoPIP2;2 proteoliposomes, and (**B**) antioxidant activity of free resveratrol extract and encapsulated extract after storage for 15 and 30 days. Data are mean ± SE (n = 3). Different letters in (**A**) indicate significant differences according to one-way ANOVA followed by Tukey-HDS test (*p* < 0.05). Different letters in (**B**) indicate significant differences among different days for each sample according to one-way ANOVA followed by Tukey-HDS test (*p* < 0.05), and asterisks (*) indicate significant differences between both empty liposomes and BoPIP2;2 proteoliposomes and free resveratrol extract according to Student *t*-test (* *p* < 0.05, ** *p* < 0.01). (**C**) Docking of resveratrol to the outer face of aquaporin tetramer showing multiple binding conformations (numbers 1–13). Resveratrol carbon backbone is shown in green; the conformation of lowest free energy of binding is represented in spheres and the rest in sticks. Aquaporin chains are depicted in green, cyan, magenta, and yellow for A, B, C, and D chains, respectively. In light blue sticks, Cys69 residues are represented, forming disulfide bridges. (**D**) Close-up of the interaction region of the docking conformation of the lowest energy of binding (pose 1 in (**C**)). Resveratrol carbon backbone is orange, and the amino acid residues are colored as their corresponding chains. Interaction distances (Å) are in dashed lines.

Resveratrol extract was encapsulated in empty liposomes and BoPIP2;2 proteoliposomes to assess the effect of protein incorporation on entrapment efficiency (EE). BoPIP2;2 was chosen for its high production efficiency and functionality. Various parameters were measured for the encapsulated extract in both liposomes and BoPIP2;2 proteoliposomes (Table 2). BoPIP2;2 proteoliposomes exhibited a 2.25-fold increase in EE compared to liposomes. As for size and PDI, these values were higher for BoPIP2;2 proteoliposomes containing the encapsulated extract. The antioxidant activity did not show differences between the free resveratrol extract and the extract encapsulated in both empty liposomes and proteoliposomes. The EE remained stable for 30 days, regardless of whether the extract was encapsulated in liposomes or proteoliposomes (Figure 3A). In terms of antioxidant activity, there was a decrease observed after 30 days of storage; however, the activity was higher when the resveratrol extract was encapsulated in liposomes and when it was encapsulated in proteoliposomes (Figure 3B).

### 2.5. Molecular Docking of Resveratrol and Integrin with PIP2 Aquaporin

A molecular docking study was performed to investigate the potential role of AQPs in the increased percentage of resveratrol encapsulation in liposomes when AQPs are included in the formulation. The aim was to elucidate if AQPs have binding sites for resveratrol, the target molecule in this study. The results of this *in silico* study revealed multiple binding conformations between resveratrol and AQP (Figure 3C). Table 3 presents a summary of all poses and the AQP residues involved in the interaction. Among the different poses, one was found in the central pore formed by the four monomers of AQP in the membrane, and this pose exhibited the lowest binding energy (−5.58 kcal/mol). The entrance to this pore is blocked by two disulfide bridges between CYS69; however, resveratrol could be located next to a disulfide bridge in a gap formed in the structure (Figure 3D). The residues contributing to this conformation were identified in several monomers of the protein, namely GLU65A, CYS69A, SER71A, and SER71C, where A and C represent different protein monomers (Figure 3D).

**Figure 4:**
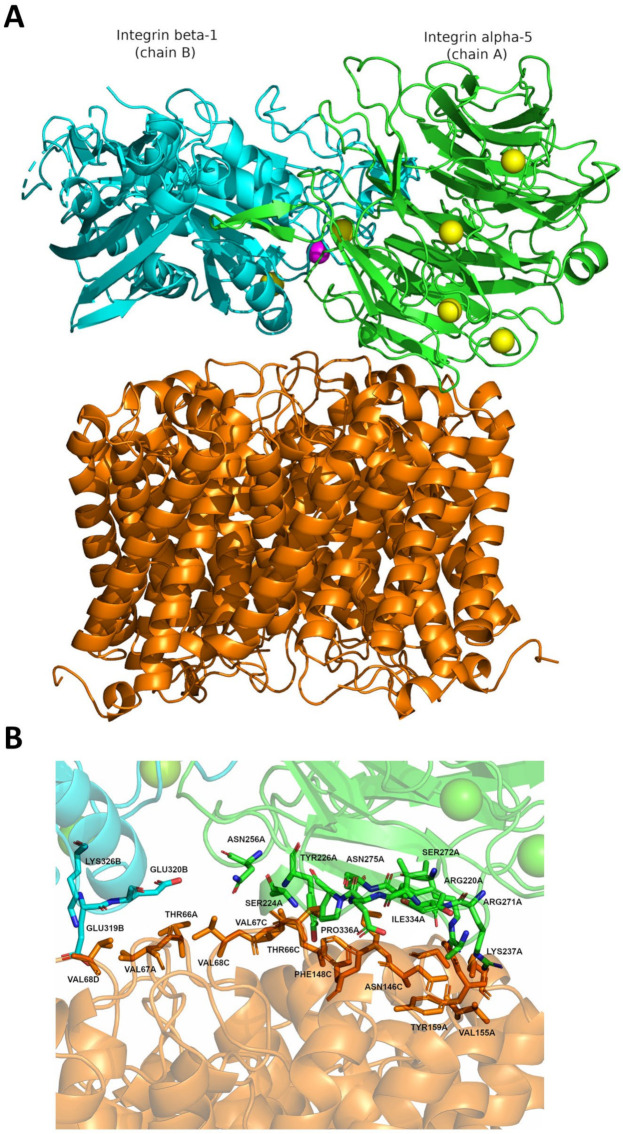
Integrin-aquaporin docking. (**A**) Docking of Integrin-Aquaporin complex showing the best scoring docking pose obtained from HADDOCK server (score = −373.70). The predicted free energy of binding calculated was −10.4 kcal/mol, corresponding to a Kd = 24 nM. Aquaporin is shown in orange (chains A, B, C, and D), and integrin in green (chain A, or Integrin alpha-5) and blue (chain B, or Integrin beta-1). Metal ions are shown as spheres, with Mg^2+^ in magenta and Ca^2+^ in yellow. (**B**) Close-up of the interaction region of the docking conformation. The amino acid residues are colored as their corresponding chains (**A**).

On the other hand, in silico modeling was performed between plant PIP2 aquaporin and human integrin, and a 3D representation is depicted in Figure 4. The best binding conformation exhibited a free energy of binding of −10.4 kcal/mol, corresponding to a Kd of 24 nM. The residues of both proteins involved in the binding are summarized in Table 4. The interaction primarily occurs between the alpha-5 integrin (A-chain) and two AQP monomers (A and C).

## 3. Discussion

AQPs are pivotal in facilitating water transport through biological membranes, holding significance for diverse biological processes [1]. Despite significant progress in understanding AQPs, many aspects of their regulation and functions remain unclear. In-depth investigations using in vitro assays with pure proteins have provided valuable insights into their mechanisms and properties [12]. The production of large quantities of pure proteins is of great interest, particularly from a physiological perspective. Pure proteins are essential for crystallography studies to determine the three-dimensional structure of proteins, shedding light on their functional mechanisms. Additionally, they are crucial for studying the functionality of transmembrane transporters or channels, such as AQPs, through reconstitution in artificial liposomes. Moreover, the production of pure proteins holds significant promise in the biotechnology industry. One notable application is in the development of devices and technologies aimed at enhancing water filtration and purification processes [13] or for the development of products with moisturizing and stabilizing properties. Heterologous expression has proven to be the most efficient method for obtaining pure proteins. Obtaining proteins from natural sources results in poor yields due to low expression levels and protein loss during purification. Challenges intensify when purifying specific AQP isoforms due to their numerous isoforms. For instance, in broccoli, more than 60 AQP genes have been described with specific but overlapping expression patterns [18]. *P. pastoris* has emerged as a superior host for recombinant protein expression compared to *E. coli*, particularly for membrane proteins. As a eukaryote, *P. pastoris* ensures proper folding and post-translational modifications [7].

In our investigation, we optimized the production protocol for BoPIP1;2 and BoPIP2;2 proteins from *B. oleracea* using *P. pastoris*. We focused on enhancing translation initiation by replacing the start codon ATG [19] with the sequence aaaATGtct, known for its suitability in yeast expression systems [20]. Furthermore, we screened clones at different zeocin concentrations to identify those with the highest gene dosage, as gene dosage correlates with protein production [10]. Zeocin concentrations of 500 µg mL^−1^ displayed the best protein expression for *BoPIP1;2*, as shown by Nordén et al. [10] for *SoPIP1;2*. In the case of *BoPIP2;2*, we selected clones with higher expression at 1000 µg zeocin mL^−1^, similar to previous studies with other human and plant AQPs [10]. These results underscore the importance of protocol optimization in attaining high protein yields and provide valuable insights for future studies on AQP expression in heterologous systems. Controlled growth is crucial for optimization, with monitored conditions in fermenters being ideal for large-scale production. An effective purification approach is also vital to sustain high yields, and detergent screening is imperative to obtain functional proteins. In this study, OG was chosen as the best option after n-dodecylphosphocholine (FC-12), which is considered a harsher detergent with a higher risk of compromising the fold of the protein of interest. OG is commonly used for solubilizing AQPs due to their stability in glucopyranosides, as observed with AtPIP2;4 [12] or SoPIP2;1 [11]. From yeast overexpressed BoPIP1;2, 0.14 mg g^−1^ of pure protein were obtained, and for BoPIP2;2, the yield was even higher, 0.99 mg per gram of yeast cell. These yields are consistent with the production range of 0.1–0.5 mg of pure protein per gram of yeast cell reported [21].

AQPs were reconstituted into liposomes, which have been extensively investigated from various perspectives, including cell membrane science, membrane proteins, and as carriers for bioactive compounds. Regarding functionality, BoPIP1;2 exhibited similar water transport to control liposomes, while BoPIP2;2 displayed a two-fold higher Pf. Similar behavior has been observed in previous studies, such as AtPIP2;4 or SoPIP2;1 reconstituted in liposomes [12] or VvTnPIP2;1 and VvTnPIP2;3 expressed in yeasts [22]. Conversely, PIP1 has been known to exhibit limited water transport capabilities. These varying results indicate that multiple factors influence the functionality of PIP1, including lipid composition, pH, and heterotetramerization with other AQPs [23]. However, in this work we can only report that BoPIP1;2 does not increase transport water (compared to control without AQPs) in our experimental conditions, and other tests would have to be performed to rule out this functionality since, as we have indicated, different factors affect water permeability. However, the fact that PIP1–PIP2 pairs were described as functional units, giving PIP2 a function of the water channel modulator [24], could endorse our results.

Considering the potential biotechnological applications of AQPs, investigating protein aggregation becomes a challenge [25]. In the stability assay, it was observed that the size and PDI of the proteoliposomes, compared to the liposomes, remained unaffected except when stored at 4 °C. Although protein aggregation typically correlates with higher temperatures, it can occur at near 0 °C, with both types following similar unfolding mechanisms [26]. Besides protein aggregation, fusion between proteoliposomes mediated by AQPs’ interaction, forming larger vesicles, should be acknowledged. Moreover, proteoliposome functionality is crucial to consider in stability assessment. The Pf of BoPIP2;2 proteoliposomes remained unchanged when stored at 4 °C and 20 °C, but a decrease in Pf was observed after storage at 37 °C, reaching a level comparable to that of the control liposomes. Thus, changes in size as well as homogeneity and retained function must be considered when finding optimal storage conditions.

The utilization of AQP proteoliposomes offers a promising strategy for enhancing the stability and bioactivity of unstable bioactives, like resveratrol-enriched grape extract, with potential applications in pharmacy and cosmetics [27]. Achieving higher EE is crucial for improved cargo absorption and bioavailability [28]. Our study revealed a 2.25-fold higher EE of resveratrol extract in BoPIP2;2-containing proteoliposomes compared to liposomes, remaining stable after 30 days, and considering that without extract there is no significant difference in the size of liposomes and BoPIP2;2 proteoliposomes. This might result from direct interactions between resveratrol molecules and AQPs. This hypothesis is supported by results obtained from molecular docking assays, which indicate potential binding sites between the PIP2 protein and the resveratrol molecule, with the most probable interaction occurring at the central pore of the AQP tetramer. Similar interactions between proteins and resveratrol have been reported in other studies [29]. Moreover, AQPs have been demonstrated to interact with different molecules and stabilize them in vitro, such as glucosinolate, as shown by molecular docking electrostatic, hydrogen bonding, and non-polar interactions [3]. Thus, BoPIP2;2 likely plays a significant role in the entrapment of resveratrol, although in addition to the interaction with AQP, the fact that AQP makes somewhat larger vesicles may also contribute to the higher encapsulation efficiency. Therefore, further studies are needed to investigate this aspect in more detail. It is worth noting that the docking was performed only on the extracellular surface of AQP, and an equal distribution of proteins between the inner and outer surfaces of the proteoliposomes is expected. This could be relevant in understanding the actual effects occurring under in vitro and in vivo conditions.

Determining the interactions of these liposomes with the target cells is crucial, considering the cosmetics’ pharmacological application. Our results with docking revealed the interaction between the AQPs present in our liposomes and the integrins found on human cell membranes. The possibility of this binding offers advantages, as integrins are molecules directly involved in the internalization of exovesicles, thereby potentially enhancing the absorption of the encapsulated active compounds by the target cells [30]. This interaction holds significant promise for improving the efficacy of the encapsulation system in delivering bioactive compounds to the desired target cells.

## 4. Materials and Methods

### 4.1. Recombinant Protein Overproduction

#### 4.1.1. Plasmid Construction and Cloning

pPICZB vectors with *BoPIP1;2* (GenBank-accession XM_013780569.1) and *BoPIP2;2* (XM_013767039.1) were purchased from Bionova-Cientifica S.L. (Madrid, Spain). The sequences belong to *Brassica oleraceae* L. var. *oleraceae*, which have 100% identity with the sequences of broccoli (*Brassica oleraceae* L. var. *italica*) [18]. Sequences were modified to optimize the start codon, ATG was replaced by aaaATGtct, and the original stop codon was omitted to allow a C-terminal translational fusion with the vector encoded Myc-epitope and 6 × His-tag. Flanking restriction sites were added (5′EcoRI–GAATTC and 3′NotI–GCGGCCGC). Plasmids were linearized by PmeI (GTTTAAAC) and were transformed into competent *P. pastoris* strain X-33 by electroporation according to the EasySelect™ Pichia Expression Kit Manual (Invitrogen, Waltham, MA, USA). Transformants were selected on YPDS (1% *w*/*v* yeast extract, 2% *w*/*v* peptone, 2% *w*/*v* dextrose, 1 M sorbitol) agar plates with 100 μg mL^−1^ zeocin. After five days, colonies were pooled and resuspended in YPDS medium and plated onto YPD-agar plates (100, 500, and 1000 μg mL^−1^ zeocin). eight colonies were streaked for single-cell colonies to stabilize the transformation, and five clones were analyzed and assigned IDs describing the isoform, the antibiotic level, and the clone number (e.g., BoPIP1;2:100:1).

#### 4.1.2. Small-and Large-Scale Expression

A small-scale expression screen was performed to analyze BoPIP1;2 or BoPIP2:2 content in clones selected at different antibiotic concentrations by Western-Blot (Primary-Ab; mouse anti-6xHis-tag, Clontech (Takara Bio Europe SF, Göteborg, Sweden), and secondary-Ab; polyclonal goat anti-mouse IgG alkaline phosphatase, Sigma (Merck Life Science AB, Solna, Sweden) as described by Nordén et al. [10]. A SoPIP2;1 clone was used as a reference [31]. One transformant for each construction was chosen for large-scale culture. Transformants were cultured on a large-scale using a 3 L benchtop fermenter (Belach-Bioteknik, Skogås, Sweden). *P. pastoris* pre-cultures in BMGY [10] were incubated at 30 °C and 150 rpm overnight. 150 mL of culture was added to 1.5 L of basal salt medium [32], supplemented with 6.5 mL of PTM_1_ salts [33]. When glycerol was consumed, a feed was initiated with 50% (*v*/*v*) glycerol and 1.2% (*w*/*v*) PTM_1_. After 6 h, the AQPs expression was induced with 100% methanol and 1.2% (*w*/*v*) PTM_1_ salts. After 50 h (OD_600_ of 400), cells were harvested (10,000× *g*, 24 min, 4 °C), and samples collected at different times from the fermenter were normalized to the same OD_600_ and analyzed by Western-Blot.

### 4.2. AQPs Purification from Pichia pastoris

#### 4.2.1. Membrane *Pichia pastoris* Preparation

Cells were resuspended in cold breaking buffer and broken in a BeadBeater (BioSpec Products, Bartlesville, USA) with glass beads by 12 × 30 s runs with cooling sessions. Cell debris was removed by centrifugation (10,000× *g*, 30 min, 4 °C). The crude membrane fraction was collected by ultracentrifugation (186,400× *g*, 1 h, 4 °C), and the resulting pellets were resuspended in cold buffer A [20 mM HEPES-NaOH pH 7.8, 50 mM NaCl, 10% (*v*/*v*) glycerol, 2 mM β-mercaptoethanol]. A urea membrane wash procedure was carried out [34], and protein concentration was assayed according to Bearden [35].

#### 4.2.2. Detergent Screening

Membranes were diluted with buffer A and mixed with different detergents to a final protein concentration of 2 mg mL^−1^ and a detergent concentration of 10 × critical micelle concentration (CMC) [5.3% n-Octyl-β-D-glucoside (OG), 2% n-nonyl-β-D-glucoside (NG), 0.47% n-dodecylphosphocholine (Fos-choline-12, FC-12), and 0.087% n-dodecyl-β-D-maltopyranoside (DDM) (Anatrace, Maumee, Ohio, USA)]. The non-solubilized and solubilized proteins were separated (150,000× *g*, 30 min, 4 °C) and checked through Coomassie and Western-Blot.

#### 4.2.3. Protein Solubilization and Ni-NTA Affinity Chromatography

The solubilized proteins were mixed with 10 mM imidazole and 4 mL of Ni-NTA agarose (Qiagen, Hilden, Germany) preequilibrated with buffer A + 3 × CMC OG and incubated overnight at 4 °C. Ni-NTA agarose with proteins was packed into empty PolyPrep-columns (Bio-Rad, Tokyo, Japan) and washed with 10-bed volumes of buffer B [20 mM HEPES-NaOH pH 7.8, 300 mM NaCl, 10% (*v*/*v*) glycerol, 5 mM β-mercaptoethanol] with 3 × CMC OG and 30 mM imidazole. The proteins were eluted in buffer B with 3 × CMC OG and 300 mM imidazole in the first elution and 500 mM in the second. Fractions were analyzed by Coomassie and Western-Blot, and protein concentration was determined by A_280_ in Nanodrop (extinction coefficient of 46.41 M^−1^cm^−1^ for BoPIP1;2 and 46.87 M^−1^cm^−1^ for BoPIP2;2, and molecular weights of 33.73 kDa and 33.14 kDa) [36].

### 4.3. AQPs Reconstitution into Proteoliposomes

Purified AQPs were reconstituted into proteoliposomes by mixing them with *Escherichia coli* lipids (Avanti Polar Lipids, Alabaster, USA) solubilized in 5% OG. The lipid-to-protein ratio (LPR) was set at 30, and the reconstitution was performed in 20 mM Tris-HCl, pH 8.0, 100 mM NaCl, 2 mM dithiothreitol (DTT), and 0.03% NaN_3_ with a lipid concentration of 2 mg mL^−1^. The mixture was incubated with gentle mixing (10 min at RT). OG was removed with Bio-Beads (2 h of incubation). The reconstituted proteoliposomes were extruded 11 times through an extruder (Avanti Polar Lipids) using a 200-nm Whatman polycarbonate membrane. Control liposomes were made in the same manner without protein. The size and polydispersity index (PDI) were measured using dynamic light scattering (DLS) on a Malvern Zetasizer NanoZS instrument (Malvern, UK) at 25 °C (3 measurements of 13 runs). Immunoblotting against the 6xHis-tag was done to confirm the integrity of the proteins. To assess the functional characterization of both AQPs, the osmotic water permeability (Pf) was measured by stopped-flow spectrophotometry in a PiStar-180 Spectrometer at 20 °C (Applied Photophysics, Leatherhead, UK) [37]. Pf was computed according to the equation: Pf = k_exp_ V_0_/A_v_ V_w_ C_out_, where k_exp_ is the fitted exponential rate constant (Figure S2), V_0_ is the initial mean vesicle volume, A_v_ is the mean vesicle surface area, V_w_ is the molar volume of water, and C_out_ is the external osmolarity. Measurements were performed at different points (0 h, 48 h, and 1 week) and at different storage temperatures (4 °C, RT, and 37 °C).

### 4.4. Resveratrol Extract Encapsulation in Liposomes and BoPIP2;2 Proteoliposomes

*E. coli* lipids were dried with nitrogen gas, and the resulting thin film was reconstituted with PBS to a final concentration of 2 mg mL^−1^. The reconstitution process involved the addition of 1 mg mL^−1^ of resveratrol extract and purified BoPIP2;2 (LPR = 30). For the control group, the same amount of Buffer A was added to form liposomes with the extract. The solutions were sonicated for 10 min in a sonicator bath (Ultrasons P-Selecta) with *a* maximum input power *of* 100 W and a *nominal* frequency of 40 kHz. To determine the EE of *the* extract, 1 mL of each sample was pelleted by centrifugation (10,000× *g*, 30 min), and the pellet was resuspended in PBS. The content of the extract in the pellet and supernatant was measured by checking A_280_. DLS was used to determine the size and PDI. The antioxidant activity was determined using the DPPH assay [38]. *The measurement* was made at the initial time and after storage at 4 °C for 15 and 30 days.

### 4.5. Molecular Docking of Resveratrol and Integrin with AQP

Molecular docking of resveratrol (PubChem Substance and Compound Database, CID 445154) was performed on the outer surface of the AQP tetramer, whose 3D structure was taken from the Protein Databank (PDB ID: 4JC6) [39], which corresponds to the aquaporin SoPIP2 from spinach (2.15 Å). The protein structure was prepared by adding all H atoms, removing octyl β-D-glucopyranoside, mercury, and Cd ions, as well as water molecules, and selecting one tetramer (chains A–D). Gasteiger atom charges (pH 7) were assigned to both resveratrol and AQP, and rotatable bonds in resveratrol were assigned using AutoDockTools4 software [40]. Docking was performed using the AutoDock 4.2.6 suite. [40]. The Lamarkian Genetic Algorithm was chosen to search for the best conformers. The number of independent dockings was set to 1000, the maximum number of energy evaluations to 2,500,000, and the population size to 150. Grid parameter files were built using AutoGrid 4.2.6 [41]. The grid box was selected to restrict docking to the outer surface of the AQP tetramer. PyMOL 2.3.0 [42] was employed to edit and inspect the docked conformations, and Wrap-Shake [43] was employed to inspect multiple binding conformations. Molecular docking of integrin (PDB ID: 4WJK), corresponding to the crystal structure of a four-domain α5β1 headpiece fragment, was also carried out on the outer surface of aquaporin as a tetramer. Protein structure was adapted for docking. Molecular docking was done with the HADDOCK server [44]. Docking conformations were selected by the HADDOCK scoring function, ignoring those integrin conformations not located on the outer surface of AQP. The prediction of binding affinity for the selected conformation was calculated using PRODIGY [45].

## 5. Conclusions

Thus, this study successfully optimized the overexpression and purification processes of two AQPs from broccoli (BoPIP1;2 and BoPIP2;2). Among the proteins studied, PIP2 demonstrated not only higher production and purification yields but also exhibited higher water transport activity. It was observed that the presence of AQPs in the system significantly increased the EE of the extract. Furthermore, in silico experiments revealed promising AQP binding possibilities, particularly with integrins found on human cell membranes. This interaction is crucial for the internalization of proteoliposomes by target cells, suggesting potential advantages for enhancing the absorption of encapsulated active compounds. Overall, these findings advance AQP-based systems for encapsulating and delivering bioactive compounds. The study underscores AQPs’ potential in biotechnological applications, particularly in interactions with target cells to enhance encapsulated compound stability and bioavailability.

## Figures and Tables

**Table 1:** Characteristics of liposomes and BoPIP1;2 and BoPIP2;2 proteoliposomes at initial time. Size (nm), polydispersity index (PDI), rate constant (s^−1^), and osmotic water permeability (Pf, µm s^−1^). Data are mean ± SE (n = 3). Different letters indicate significant differences between conditions for each sample according to one-way ANOVA followed by Tukey-HDS test (*p* < 0.05).

	Size (nm)	PDI (0–1)	Rate Constant (s^−1^)	Pf (µm s^−1^)
Liposomes	296.95 ± 36.20 ^a^	0.34 ± 0.04 ^a^	4.21 ± 0.62 ^a^	115.75 ± 17.10 ^a^
BoPIP1;2 proteoliposomes	255.63 ± 20.62 ^a^	0.32 ± 0.01 ^a^	3.76 ± 0.25 ^a^	89.11 ± 5.93 ^a^
BoPIP2;2 proteoliposomes	278.80 ± 37.50 ^a^	0.33 ± 0.02 ^a^	9.66 ± 0.79 ^b^	249.38 ± 20.46 ^b^

**Table 2:** Physicochemical characterization of resveratrol extract in liposomes and BoPIP2;2 proteoliposomes. Entrapment efficiency (EE, %), size (nm), polydispersity index (PDI), and antioxidant activity (DPPH, µM Trolox equivalent (TE) g^−1^). Data are mean ± SE (n = 3–5). Different letters indicate significant differences between samples according to one-way ANOVA followed by Tukey-HDS test (*p* < 0.05).

	EE (%)	Size (nm)	PDI	DPPH (µM TE g^−1^)
Free resveratrol extract	/	/	/	1578.34 ± 167.27 ^a^
Liposomes	/	218.93 ± 7.99 ^b^	0.46 ± 0.02 ^ab^	/
BoPIP2;2 proteoliposomes	/	264.83 ± 8.05 ^ab^	0.53 ± 0.03 ^a^	/
Liposomes with resveratrol extract	23.17 ± 3.51 ^b^	223.10 ± 7.56 ^b^	0.22 ± 0.05 ^c^	1624.84 ± 121.88 ^a^
BoPIP2;2 proteoliposomes with resveratrol extract	52.31 ± 3.35 ^a^	358.33 ± 48.4 ^a^	0.36 ± 0.01 ^b^	1426.92 ± 118.92 ^a^

**Table 3:** Resveratrol interactions with aquaporin. The data correspond to the different docking poses of resveratrol in Figure 3. The free energy of binding (ΔG) and the dissociation equilibrium constant (Kd) of resveratrol are shown.

Pose #	ΔG (kcal/mol)	Kd (nM)	Amino Acid Residues within 2.5 Å of the Ligand				
1	−5.58	80	GLU65A	CYS69A	SER71A	SER71C	
2	−5.34	120	LYS64A	LYS138A	ALA139A	LYS142A	ASN160D
3	−5.19	160	LYS64B	LYS142B	ASN160C	THR163C	
4	−4.97	230	SER154B	LYS64D	GLY70D		
5	−4.97	230	GLY61A	LYS64A	THR66A	SER154D	
6	−4.94	240	ASN160A	THR163A	ALA139C	LYS142C	
7	−4.91	250	LYS64B	GLU65B	ALA152C	SER154C	
8	−4.89	260	ASN160B	THR163B	LYS64D	ALA139D	
9	−4.87	270	VAL68A	VAL67D	CYS69D	GLY70D	
10	−4.81	300	VAL67B	CYS69B	SER71B	GLU65D	
11	−4.80	300	ALA152A	GLY218A	ARG225A	GLU65C	
12	−4.75	330	GLU65B	GLU65C	VAL67C	GLY70C	
13	−4.49	510	HIS62B	SER63B	PHE148B	GLY218B	ARG225B

**Table 4:** Integrin-aquaporin interactions. The data correspond to the docking pose shown in Figure 4. Amino acid residues are selected within 3.5 Å.

Integrin	Aquaporin	Distance (Å)
ARG220A	VAL155A	3.37
ARG220A	LYS237A	1.71
SER224A	GLN147A	2.18
TYR226A	VAL67C	3.42
ASN256A	VAL68C	3.29
ARG271A	VAL155A	3.37
ARG271A	GLY158A	3.21
ARG271A	TYR159A	2.71
ARG271A	LYS237A	3.00
SER272A	GLY158A	2.49
TYR274A	GLY143C	3.46
TYR274A	GLN147C	2.86
ASN275A	THR66C	2.43
ASN275A	GLN147C	3.24
ALA332A	ASN146C	2.35
ILE334A	GLN147C	2.71
GLU335A	ASN146C	3.03
GLU335A	GLN147C	2.75
PRO336A	GLN147C	2.71
PRO336A	PHE148C	3.48
GLU319B	VAL67A	3.08
GLU320B	THR66A	3.33
LYS326B	VAL68D	2.56

## Data Availability

The original contributions presented in the study are included in the article/Supplementary Materials; further inquiries can be directed to the corresponding author/s.

## Supplementary Materials

The supporting information can be downloaded at: https://www.mdpi.com/article/10.3390/ijms25041987/s1.
