# Supplementary material for: Brassica oleracea L. var. italica Aquaporin Reconstituted Proteoliposomes as Nanosystems for Resveratrol Encapsulation
Source: Int J Mol Sci. 2024 Feb 6;25(4):1987. doi: 10.3390/ijms25041987 (PMC10888208; doi:10.3390/ijms25041987)
Supplement: Supplementary file 1 [file ijms-25-01987-s001.zip › ijms-2849302-supplementary.pdf]

Figure S1

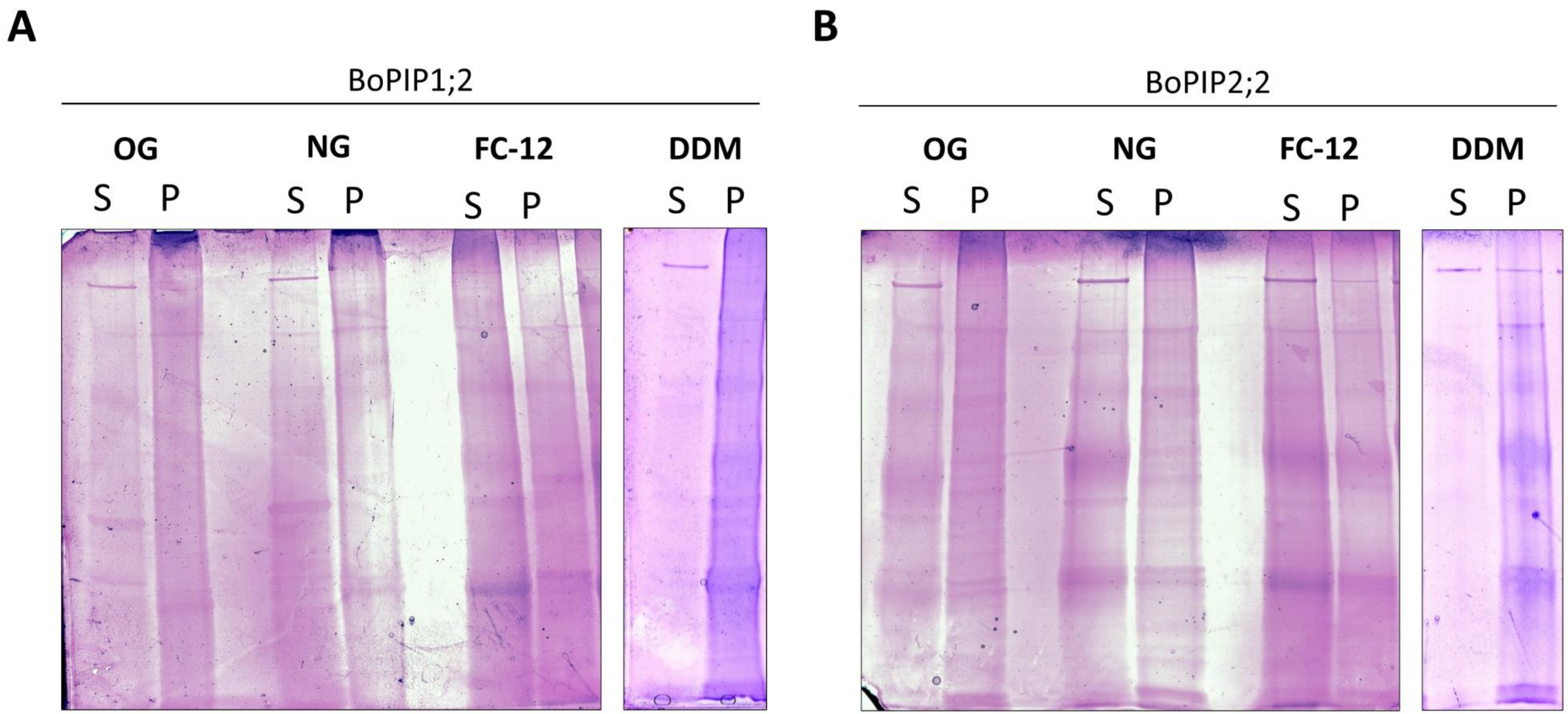

Figure S1.- Detergent screen. Coomassie stained SDS-PAGE gels with the solubilized supernatant (S) fractions and the unsolubilized pellet (P) fractions. Four detergents (n-Octyl-β-D-glucoside (OG), n-nonyl-β-D-glucoside (NG), n-dodecylphosphocholine (Fos-choline-12), and n-dodecyl-β-D-maltopyranoside (DDM)) were screened.

Figure S2

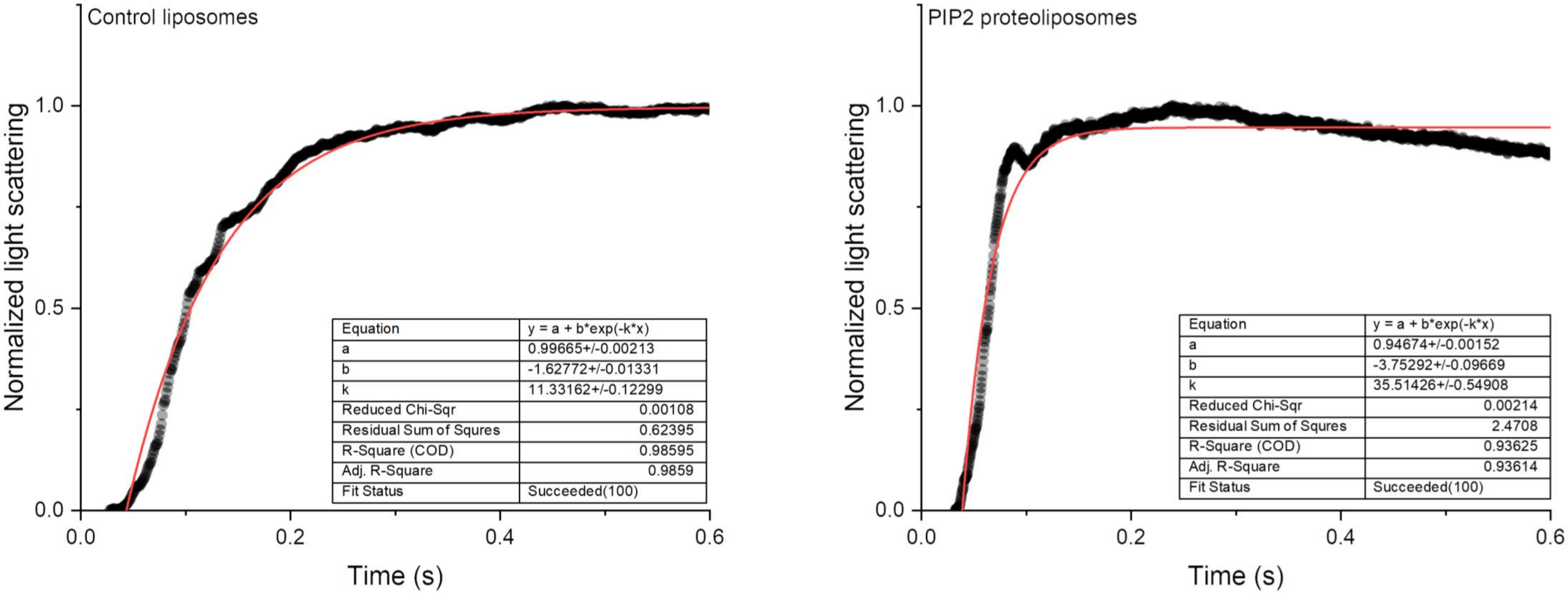

Figure S2.- Time course of increased scattered light intensity in control liposomes and PIP2 proteoliposomes (black) introduced in a stopped-flow apparatus; for both types of membrane preparation, a typical experiment and the fitted monoexponential function are shown.
